# Toward a Detailed Description of the Thermally Induced Dynamics of the Core Promoter

**DOI:** 10.1371/journal.pcbi.1000313

**Published:** 2009-03-13

**Authors:** Boian S. Alexandrov, Vladimir Gelev, Sang Wook Yoo, Alan R. Bishop, Kim Ø. Rasmussen, Anny Usheva

**Affiliations:** 1 Theoretical Division and Center for Nonlinear Studies, Los Alamos National Laboratory, Los Alamos, New Mexico, United States of America; 2 Beth Israel Deaconess Medical Center, Harvard Medical School, Boston, Massachusetts, United States of America; University of Houston, United States of America

## Abstract

Establishing the general and promoter-specific mechanistic features of gene transcription initiation requires improved understanding of the sequence-dependent structural/dynamic features of promoter DNA. Experimental data suggest that a spontaneous dsDNA strand separation at the transcriptional start site is likely to be a requirement for transcription initiation in several promoters. Here, we use Langevin molecular dynamic simulations based on the Peyrard-Bishop-Dauxois nonlinear model of DNA (PBD LMD) to analyze the strand separation (bubble) dynamics of 80-bp-long promoter DNA sequences. We derive three dynamic criteria, bubble probability, bubble lifetime, and average strand separation, to characterize bubble formation at the transcriptional start sites of eight mammalian gene promoters. We observe that the most stable dsDNA openings do not necessarily coincide with the most probable openings and the highest average strand displacement, underscoring the advantages of proper molecular dynamic simulations. The dynamic profiles of the tested mammalian promoters differ significantly in overall profile and bubble probability, but the transcriptional start site is often distinguished by large (longer than 10 bp) and long-lived transient openings in the double helix. In support of these results are our experimental transcription data demonstrating that an artificial bubble-containing DNA template is transcribed bidirectionally by human RNA polymerase alone in the absence of any other transcription factors.

## Author Summary

Accessing the information encoded in DNA requires that RNA polymerases recognize the core promoter, a sequence that marks the start of a gene. Statistical analysis of known promoter sequences has failed to reveal a simple code for identifying promoters, leading to the suggestion that promoter DNA is distinguished by certain structural/dynamic properties encoded in nonobvious ways by the literal sequence. Because the DNA strands at the promoter need to be separated for transcription to begin, we previously proposed that promoter sequences exhibit a propensity for spontaneous strand separation. Here, we conduct simulations of the ultrafast, small-scale strand separation motions of eight mammalian promoters and show that start sites tend to form larger and more stable openings in the double helix compared to other sequences. Experimentally, we show that an artificial permanent opening in the double helix is sufficient for transcription in the absence of sequence-specific protein–DNA contacts. These findings support a view of DNA as a structurally active participant in gene expression, rather than the commonly envisioned passive digital storage device. Our analysis suggests that functionally relevant structural variation in genomic DNA occurs at the level of fast motions not readily observed by traditional molecular structure analysis.

## Introduction

It is generally acknowledged that the structure and dynamics of DNA at the eukaryotic promoter play important roles in gene regulation, but the nature of this relationship is unclear. From a structural perspective, RNA polymerases require single stranded DNA, or the formation of a ‘transcriptional bubble’ at the transcriptional start site (TSS) to initiate transcription [1],[2]. Eukaryotic transcription initiation often proceeds from a negatively supercoiled template in the absence of helicases [3]–[6], implicating spontaneous local melting of dsDNA as a key feature of promoter sequences. Furthermore, introduction of few mismatched bases to unzip the DNA at the start site allows transcription in the absence of supercoiling [6],[7]. It is likely that locally enhanced breathing dynamics of the DNA are a common feature of the TSS, required to seed the formation of the transcriptional bubble. We previously showed a correlation between transcriptional start site location, single strand nuclease sensitivity, and transient dsDNA strand separation predicted by statistical calculations with the nonlinear Peyrard-Bishop-Dauxois (PBD) model of DNA [8],[9]. This one-dimensional model, originally designed to explain DNA melting profiles, has successfully reproduced thermodynamic parameters for DNA phase transitions [10], helicase unwinding force calculations [11], mechanical unzipping [12] and DNA bubble nucleation experiments [13]. Statistical thermodynamic implementations of PBD are fast enough to allow recently the calculation of the local melting (bubble) probability profile of the entire Adenoviral genome (30 Kb) [14]. Such calculations, however, require pre-defined bubble size thresholds and yield probability values that contain no information about bubble lifetimes and the frequency of DNA breathing motions. In search of the distinguishing dynamic features of gene promoter TSS sequences, we performed PBD-based Langevin molecular dynamic (LMD) simulations [8],[15] of eight experimentally characterized mammalian core promoters. From the LMD trajectories we extracted three distinct dynamic characteristics: bubble probability, bubble lifetime, and the average strand separation coordinates. The calculated dynamical profiles suggest that a relatively large, long-lived DNA bubble commonly forms at the transcription start site.

## Methods

### The Peyrard-Bishop-Dauxois (PBD) Model

The PBD model is a one-dimensional nonlinear model that describes the transverse opening motion of the opposite strands of dsDNA. The Hamiltonian of the model is
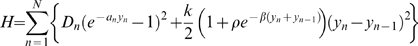
(1)where the sum is over all N base pairs of the DNA. *y_n_* denotes the relative displacement from equilibrium of the complementary bases of the *n*-th base pair, divided by √2. The first term of the Hamiltonian is the Morse potential which represents the base pair hydrogen bonds together with the electrostatic repulsion of the backbone phosphates. The parameters *D_n_* and *a_n_* depend on the nature of the base pair (A-T vs. G-C) at site *n*. The second term represents a harmonic potential approximation but with a nonlinear coupling constant, which takes into account the influence of the stacking interactions between consecutive base pairs on the transverse stretching motion. The exponential term effectively decreases the harmonic spring constant K when one of the base pairs is displaced away from its equilibrium position in the double helix: K_max_ = k (1+ρ); when *y_n_*+*y_n−1_* = 0, a condition met, e.g., at equilibrium, and K_min_ = k; when *y_n_* or *y_n−1_*→***∞***, i.e., when at least one of the base pairs is out of the double helix stack. This term is essential for simulating long-range cooperative effects important for sharp DNA melting [16]. The parameters of the model have been previously obtained by fitting simulations to DNA UV melting curves [10].

### Langevin Dynamic Simulations

**Figure 1:**
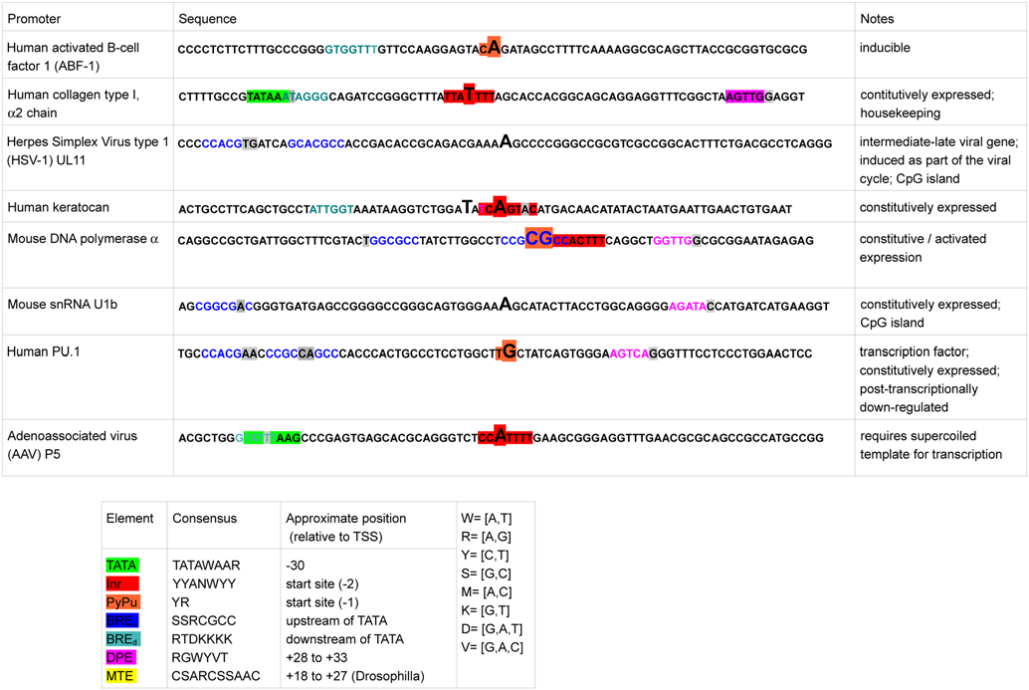
Core promoter sequences analyzed by PBD Langevin dynamics simulations. Experimentally verified transcriptional start sites (TSS) are shown in large letters. Common promoter sequence elements are indicated by colored boxes. For illustrative purposes, sequences that fit the element definitions but are not properly positioned relative to the TSS are also shown as colored letters. Deviations from the consensus sequence are indicated in gray. The sequences were obtained from the Eukaryotic Promoter Database (EPD, http://www.epd.isb-sib.ch/). The identity of each promoter is described in column 1, the sequence is shown in column 2, and the mode of regulation in column 3.

Langevin molecular dynamics simulations were performed at *T* = 310 K, by numerically integrating systems of stochastic equations based on the Peyard-Bishop-Dauxois (PBD) model. Periodic boundary conditions were applied in order to avoid terminal base pair effects, effectively circularizing the DNA sequence (but without any torsional effects). Each DNA sequence (Figure 1) was simulated in 1000 separate realizations for 1 ns, using 1 fs timesteps and a 200 ps preheating time. Simulations were performed on Linux clusters at LANL and Harvard Medical School.

### Analysis of the Dynamic Trajectories

**Figure 2:**
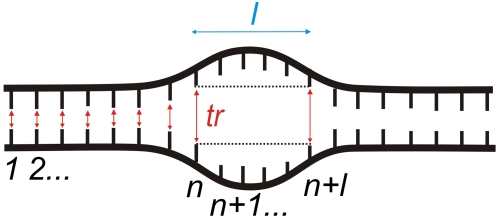
Schematic representation of a DNA bubble with length *l* [bp] and amplitude *tr* [Å] at postition *n*.

The probability P_n_ *(l,tr)* for the existence of a bubble (collective opening) of a certain length *l* base pairs and amplitude threshold (*tr*, Å) (Figure 2) [15] was calculated as
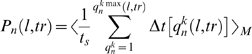
(2)where < >*_M_* denotes averaging over M simulations and *t*_s_ is the time of the simulation. *q^k^_n_*(*l*, *tr*) enumerates the bubbles of duration Δt[*q^k^_n_*(*l*, *tr*)] with amplitude *tr* [Å] and length *l* base pairs, beginning at the *n* ^th^ base pair in the *k* ^th^ simulation.

The average bubble duration τ*_Lifetime_* was calculated as the average lifetime of a bubble of a given shape, i.e., with amplitude *tr* [Å] and length *l* [bp], over all occurrences of that bubble.
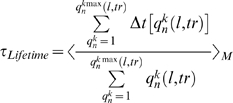
(3)


### Average Coordinate Calculations

The average displacement of each base pair from its equilibrium double stranded conformation was calculated for the adeno-associated virus P5 promoter in two ways: using Metropolis Monte Carlo algorithm [13] and by averaging over all Langevin dynamics trajectories obtained in the above MD simulations.

### Bubble Shape Calculations

The average lifetime of all bubbles (see Eq. 3) of a given shape, i.e., with amplitude *tr* [Å] and length *l* [bp] *containing* a given base pair was calculated from the Langevin dynamic trajectories, and plotted as a function of bubble length and bubble amplitude.

### Transcription Reactions

The sequence of the DNA promoter template, assembly of the run-off transcriptional reactions, purification of human RNA polymerase II, RNA product separation, and visualization have been previously described [6]. The control nonpromoter sequence (80 bp) is part of the published sequence for the human collagen intron (NW_927317) GCAAACGCCGTCGTCCGCACCGGTCGCGACTCGGCAAGGGAGCGGGCGGAAGCTGACTCG CGGCGGAGG GGGGTCACTC.

All figures are assembled using Photoshop, FreeHand, Mathematica and MATLAB.

## Results/Discussion

For this study we chose a set of mammalian gene promoters with experimentally verified transcriptional start sites and presumably diverse mechanisms of regulation (Figure 1). The group includes constitutively expressed, inducible, viral, and transcriptional regulator core promoters. To ensure diversity, the chosen promoters contain various combinations of promoter elements, DNA sequences commonly found at core promoters (reviewed in [17],[18]). Langevin simulations were performed on 80–100 bp sequences centered at the transcriptional start site (TSS) and the trajectories from 1000, 1 ns simulations were analyzed to extract different features of thermally-induced dynamics of DNA strand separation (Figure 2). The probability of collective opening to form a bubble at a given site was determined from the lifetimes of all open states above a given length and amplitude, normalized over the time of the simulation (Eq. 2). Bubble lifetimes were calculated by averaging the duration of an opening with given amplitude and given length over all occurrences of that opening (Eq. 3).

### Bubble Probability

**Figure 3:**
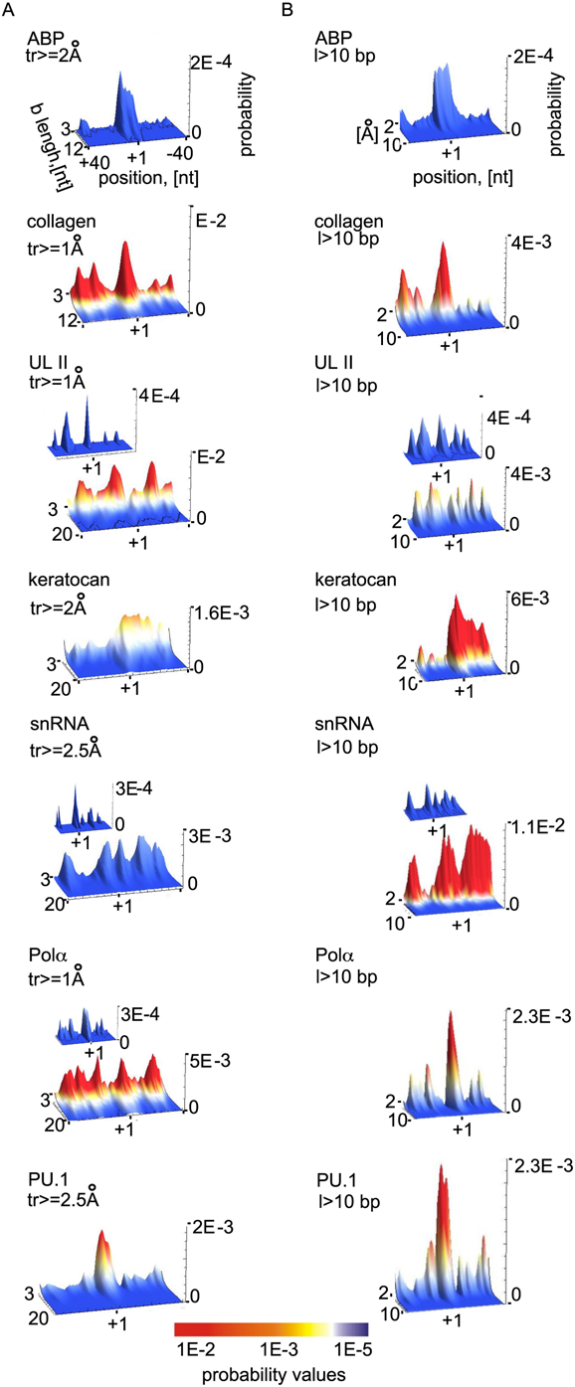
Probability for DNA collective openings of mammalian core promoters, calculated from PBD Langevin dynamic simulations. The probability was determined from the lifetimes of all open states above a given length and amplitude, normalized over the time of the simulation (Eq. 2). (A) Probability for opening (vertical axis) starting at specific nucleotide positions (horizontal axis), as a function of bubble length [bp]. Probability values are colored to the same scale between promoters for comparison. Nucleotide positions are labeled relative to the TSS (+1). Promoter identity and bubble amplitude thresholds are shown at the top. The thresholds are chosen individually for each promoter, as the smallest values for which the TSS region begins to exhibit maximum probability. (B) Probability for opening (vertical axis) starting at specific nucleotide positions (horizontal axis), as a function of bubble amplitude [Å]. Probability values are colored to the same scale between promoters for comparison. Nucleotide positions are labeled relative to the TSS (+1). Promoter identity and bubble length thresholds are shown at the top of the panels. The thresholds are chosen individually for each promoter, as the smallest values for which the TSS region begins to exhibit maximum probability.

Figure 3 shows the probability for the formation of bubbles above a certain amplitude *tr* as a function of bubble length (Figure 3, panel a), as well as above a certain length *l* as a function of amplitude (Figure 3, panel b). The observed profiles differ significantly between promoters, both in probability values (color scale) and overall peak distribution, especially when bubbles of any size are considered (not shown). However, bubble length *l* (panel a) and strand separation amplitude values (panel b) can be found for each promoter, above which the TSS displays the maximum probability. These thresholds vary between promoters, but in all cases except the HSV UL11 and snRNA, bubbles longer than 10 bp and with larger than 2 Å amplitudes are most likely to be present at the TSS. In comparison, the UL11 and snRNA promoters are very active across the entire simulated promoter segments, and the TSS only become predominant for very large bubbles (panels a, b insets). The human ABF-1 promoter is the least dynamically active, with bubbles of *l*>10 bp and *tr*>1 Å (panel b), an order of magnitude less likely than similar size bubbles in the other promoters, but a very well pronounced TSS bubble.

Overall, the probability for the occurrence of bubbles longer than 10 bp varies between ∼10^−4^ and ∼10^−3^ for bubbles with larger than 1 Å amplitudes, and is in the order of 10^−5^ for *tr*>3 Å. Interestingly, NMR studies estimated comparable probabilities (∼10^−5^) for single base pair openings that lead to exchange between base paired hydrogens and water [19]. Comparison between the probability plots and the promoter element distributions (Figure 1) reveals intriguingly that ‘classic’ promoters that contain well-known sequence motifs exhibit ‘clean’ dynamic profiles with strong peaks at the TSS, while the dynamic profiles of two promoters without known elements have poorly defined start site bubbles. Such difference could arise from higher G/C content of these two promoters, causing a bias in the simulations, as discussed in the last section. Alternatively, the observed probability differences may reflect differences in transcriptional regulation.

### Bubble Lifetime

**Figure 4:**
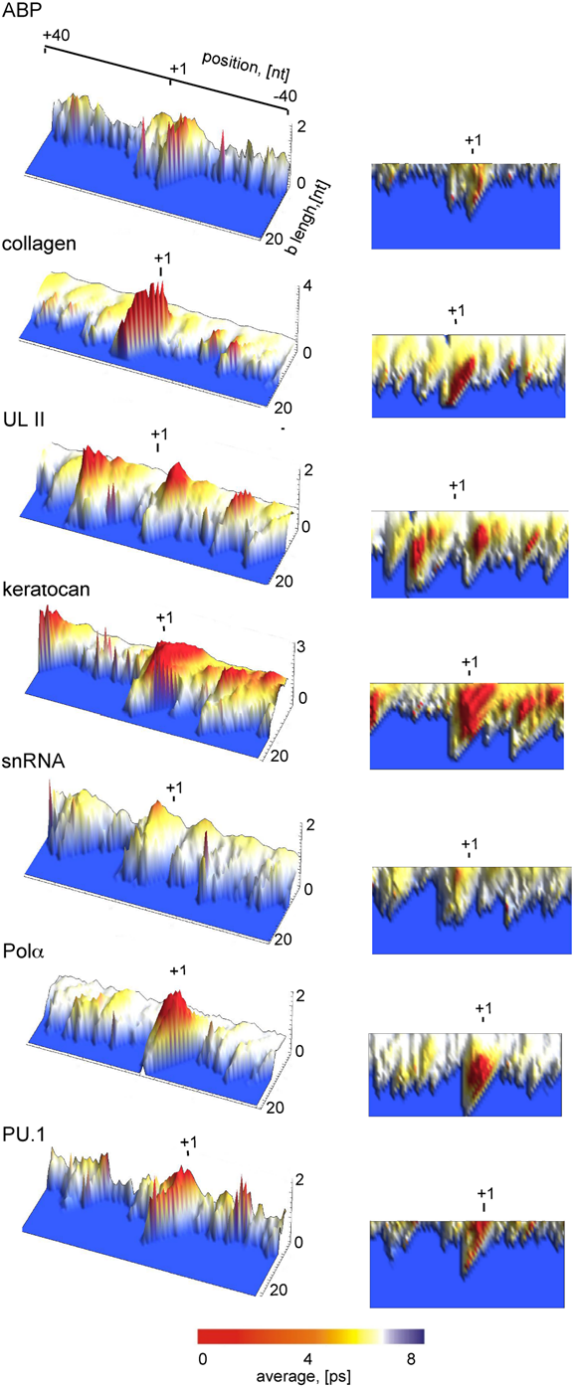
Average lifetimes of DNA collective openings of core promoter sequences, as a function of length[bp]. For clarity, the same promoter profiles are shown from a different angle in the panels at the right. Nucleotide positions are shown relative to the TSS (+1). The TSS is marked with a vertical line. The color scale represents the average lifetimes [ps]. The identity of the promoters is shown above the panels.

To further characterize the DNA dynamics of the selected promoters, we used the simulated Langevin trajectories to derive the average lifetime of a given opening as a function of base pair length and amplitude (Eq. 3). Figure 4 shows the lifetimes of bubbles above certain amplitude, as a function of bubble length. The bubble lifetime profiles are more closely related among the studied promoters than the probability profiles (Figure 3). The longest-lived openings are clearly present at the transcriptional start site in most cases. Exception is again the mouse snRNA promoter, where the TSS is only slightly predominant as well as the UL11 promoter, where bubbles of similar size and stability are also present 25 bp up- and downstream of the TSS. Overall, the most stable bubbles are ∼10 bp long, with the exception of the snRNA promoter (5 bp). A notable feature of the plots is that in some cases longer bubbles are significantly more stable than smaller ones at the same location.

As previously pointed out in the literature [14],[15],[20],[21], statistical probability calculations do not always predict the most likely opening to be at the TSS, and regulatory sites 20–30 bp up- or downstream of the TSS, such as a TATA box often exhibit a higher probability for opening that the start site in such calculations. In the present study, the probability for strand separation of the collagen promoter is similar at the TATA box region and the transcription start site (Figure 3), but a remarkably stable (5 ps) concerted opening of 10–15 bp is seen *only* at the TSS (Figure 4). In contrast, the UL11 promoter displays three bubbles that are similar both in terms of probability and lifetime, at the TSS and flanking regions. According to our results the TSS and TATA-box in the collagen promoter exhibit distinct dynamic behavior. Namely, the TSS displays a lower frequency of opening but forms relatively stable bubbles, while the TATA-box region is characterized by higher frequency motions, forming bubbles of low duration.

**Figure 5:**
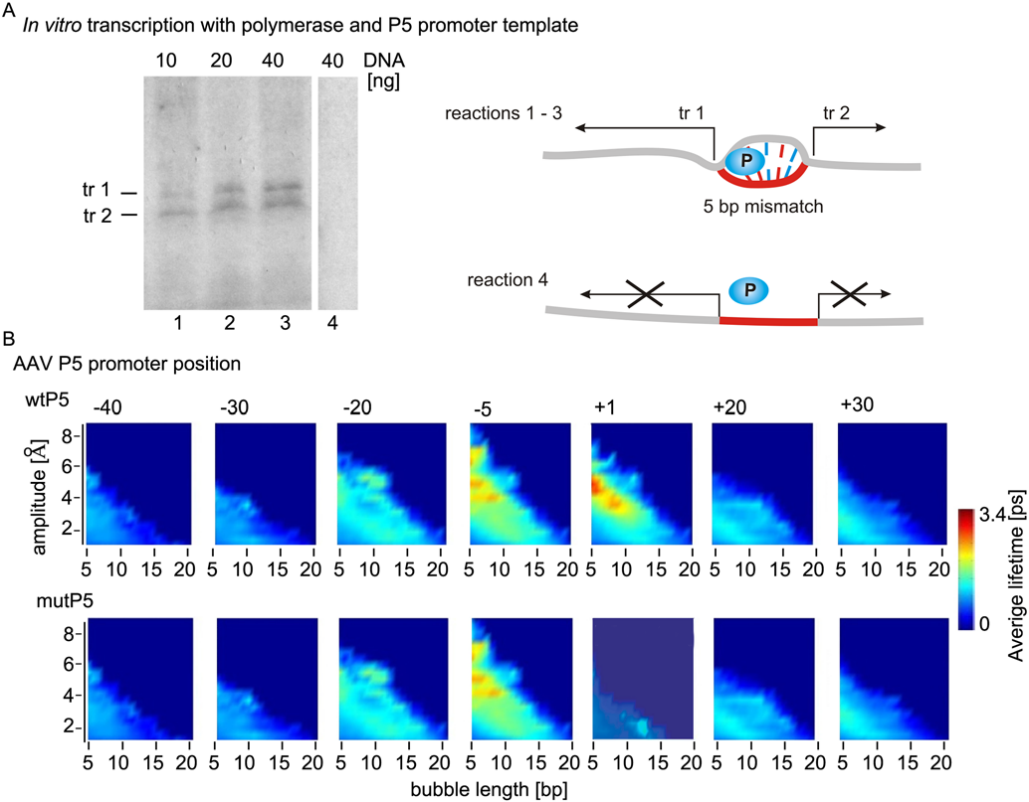
Supercoiling and artificial mismatch bubbles enable transcription from the P5 promoter according to the Usheva, Shenk (1996) experiment. (A) Artificial mismatch bubbles enable bidirectional transcription from the P5 promoter by human PNAP2 in the absence of transcription factors. All reactions received 2 units of purified RNAP2 and different amount of synthetic linear ds DNA template with the AAV P5 promoter as indicated at the top of the lines. The DNA template in reactions 1, 2, and 3 contains 5 bp long mismatches creating a “bubble” in the region of the transcription start site. The reaction in line 4 received ds DNA with no mismatch. The ^32^P- labeled reaction RNA products have been separated by gel electrophoresis based on difference in the size of the transcripts. The position of the specific RNA transcripts is shown on the left: tr1- transcripts that initiate at the bubble and terminates at the 5′-prime end of the DNA template; tr2 – transcripts initiated at the bubble and terminated at the 3′-end of the template. The migration of DNA size markers was used to determine the position of the specific transcripts (not shown). Schematic diagram of the experiment is presented at the left. The bidirectional transcription from the mismatched DNA template (gray) is labeled with black arrows. The promoter region is labeled with red and the polymerase with blue (P). (B) Bubble lifetime as a function of length and amplitude at 310°K, shown for individual base pairs of both, the wild type (wt) P5 and the mutant (mt) P5 variant. Each square presents the average lifetimes (color scale) of all bubbles at a given amplitude (vertical axis) and length (horizontal axis), containing a given base pair (top right). Transcription starts at base pair +1.

As previously reported [15],[20], the adenoassociated virus (AAV) P5 promoter displays a higher probability for opening at the TATA box than at the TSS. A detailed profile of the bubble lifetimes at individual base pare promoter positions is shown in Figure 5, panel b. Analogous to the collagen promoter, bubbles around the AAV P5 TATA box again have significantly shorter lifetimes (−30, Figure 5, panel b) than bubbles formed around the TSS (+1).

The calculated bubble lifetimes (Figure 4) are in the order of few picoseconds, a number that is somewhat dependent on the choice of the PBD parameters. PBD is a phenomenological representation of DNA melting behavior, and water collisions are implicitly modeled in the Langevin simulations, necessarily yielding a qualitative description of dynamic lifetimes. Our focus here is therefore on relative but not absolute timescales.

### Dynamics of Nonpromoter Sequences

**Figure 6:**
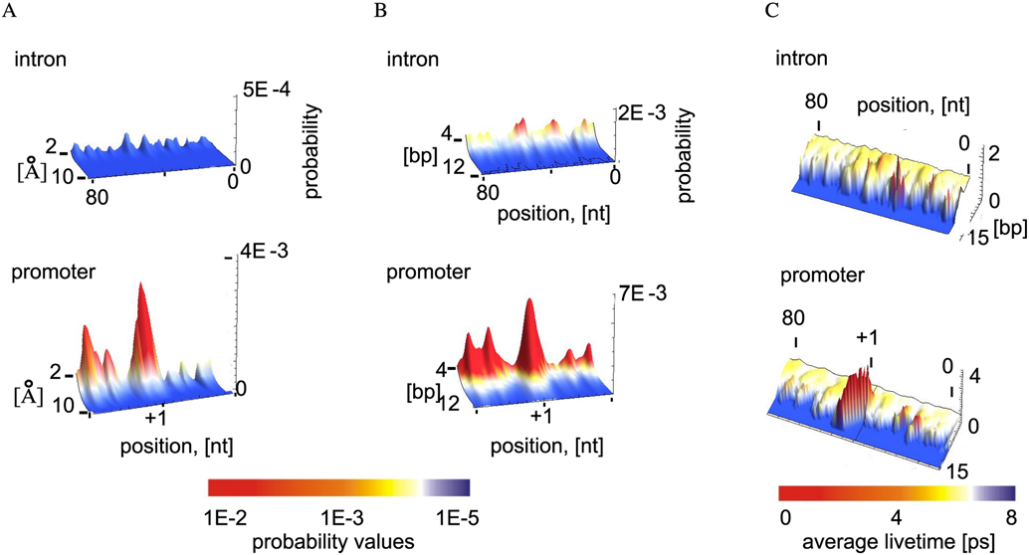
Collective opening profiles of the collagen nonpromoter sequence calculated from the PBD Langevin dynamic simulations. (A) Probability for collective opening (vertical axis) of ten base pairs starting at specific nucleotide position within the collagen intron (horizontal axis), as a function of bubble amplitude [Å]. For comparison the profile of the collagen promoter is also presented (bottom panel). Probability values are colored to the same scale between the promoter and the intron sequences, as shown below the plots. Nucleotide positions in the collagen promoter are labeled relative to the TSS (+1). The sequence identity is shown at the top. (B) Probability for opening (vertical axis) of amplitude threshold (tr)≥1 Å, starting at specific nucleotide positions (horizontal axis), as a function of bubble length [bp]. Probability values are colored to the same scale, as shown below the plots. The sequence identity is shown at the top. (C) Average lifetimes of DNA collective openings of amplitude tr≥1 Å (vertical axis), starting at specific nucleotide positions (horizontal axis), as a function of length [bp]. The average lifetimes of collective openings for the collagen promoter are shown below. The TSS is marked with a vertical line. The color scale shown below the plots represents the average lifetimes [ps].

To verify that the observed DNA dynamic profiles are relevant to transcription initiation, we performed identical PBD-LMD simulations on nonpromoter DNA sequences. The simulation results for the intron sequence of the human collagen gene are shown in Figure 6. The intron sequence was chosen to exclude transcription factors binding sites, as we previously showed that such sites are often dynamically active (14). As shown (Figure 6) the intron sequence displays significantly lower propensity for strand separation both in terms of probability for opening with given amplitude (panel a), probability for opening with given length (panel b), and bubble lifetime (panel c). The profiles of other examined sequences containing the repeats: [ATATATATAT]_7_, [GCGCGCGCGC]_7_, [GCATGCATGC]_7_, [GCGCGATATA]_7_, [GCGATA]_12_ also lacked localized bubbles (not shown) of the size and lifetime observed for the studied core promoters.

Our data support the conclusion that nonpromoter sequences lack the characteristic signature of strand separation dynamics of the gene promoters.

### The Transcriptional Bubble

That bubbles, such as those predicted by the simulations, are coupled to biochemical DNA events is suggested not only by the successful reproduction of DNA melting [10] and unzipping [12] data by the PBD model, but also by single strand nuclease sensitivity and *in vitro* transcription experiments. We previously reported such experiments for the AAV P5 and adenoviral major late (AdMLP) promoters [8]. The role of DNA local melting in eukaryotic transcription is supported by the fact that inserting a promoter in a supercoiled plasmid allows transcription to proceed in the absence of helicase activity [3],[4], and even in the absence of the TATA box binding protein TBP in a TATA box promoter [5],[6]. Here we demonstrate that human RNA polymerase II (RNAP2) bidirectionally initiate transcription in the absence of *any* transcription factors, if an artificial long-lived bubble of >/ = 5 bp is introduced at the start site of the AAV P5 promoter (Figure 5, panel a, lanes 1, 2, and 3). When the DNA template is linear and unzipped, transcription does not proceed (panel a, lane 4), even though the promoter sequence DNA is intact (panel a, schematic diagram). These results could explain our previously reported experimental data with linear and supercoiled AAV P5 promoter DNA templates [6]. They suggest that some structural aspect of the DNA sequence is favorably enhanced by the external unwinding force of supercoiling in the promoter region. The transcriptional data here (panel a), together with the previously published results by us and also by others, clearly suggests that the aspect in question is most likely local DNA melting, remarkably enabling bidirectional transcription by RNAP2 alone. The calculated bubble lifetime profile of the P5 promoter (panel b) is consistent with the idea that a transient local bubble in the dsDNA at the promoter, amplified and stabilized by negative supercoiling, is necessary for transcription initiation by RNAP2. The role of transcription factors including YY1 in this case appears to be to further assist bubble formation, and direct the transcription reaction only downstream of the TSS [6].

### Average Strand Coordinates

**Figure 7:**
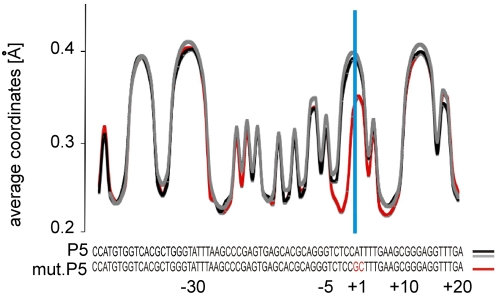
Average base pair separation coordinates for the AAV P5 promoter. Average base pair separation coordinates [Å] calculated from the Langevin dynamic trajectories of the AAV P5 promoter (black line) and a transcriptionally silent mutant (red line). For comparison, the average coordinates calculated with Monte Carlo simulations are also shown (gray line). The P5 sequence is shown under the plot. The transcriptional start site (TSS) is marked with a blue line. Mutated residues that silence transcription are shown in red letters.

Besides the statistical probability and lifetimes of the open states, the Langevin dynamic trajectories can be used to derive the average displacement of the dsDNA base pairs from their equilibrium closed state. Figure 7 shows the average displacements of bp −47 to +22 of the adeno-associated virus P5 promoter and a transcriptionally silent A>G/T>C mutant [8]. We previously reported a dramatic difference in the bubble probability at the mutated site in those two sequences [8],[15], matching the dramatic difference in transcriptional activity of the promoters. The average displacements calculated by Monte Carlo (MC) simulations are also shown for comparison with the Langevin data. The results from the LMD and MC simulations are virtually identical, as should be expected from properly conducted simulations. The strongest signals in the P5 promoter are again at the TATA box and TSS, but in contrast to the probability distributions (Figure 3), and average lifetimes (Figure 4), the average coordinates of the TATA box and the TSS do not stand out so clearly. Curiously, the simulations predict differences as large as 0.2 Å in the average base pair length at different positions of AAV P5. Such significant differences should be experimentally detectable by NMR measurement of residual dipolar couplings in a weakly oriented medium [22]. The slightly lower average displacement of the TSS region compared to the TATA box is consistent with the idea that bubbles there are formed more rarely but persist longer and have higher amplitudes. A comparison between the average displacement profiles of *wild-type* P5 promoter and the transcriptionally silent mutant (Figure 7) reveals a rather small difference in the average displacement of the TSS position, in contrast to the dramatic difference in the bubble lifetime profiles (Figure 5, panel b). This result supports the notion that bubble lifetime, probability, and average amplitude are distinct dynamic properties with nontrivial dependence on DNA sequence. The data suggest that the studied TSS are more easily distinguished by lifetime and bubble probability, than average displacement. Nevertheless, if the average strand displacements predicted here are accurate, variations of such magnitude in the double helix width may have a functional effect on protein-DNA recognition in general.

### Transcriptional Bubbles and Regulation

Despite the differences (Figure 1) in type of regulation (e.g., always turned ON ‘housekeeping’ vs. highly regulated between low and high level of expression mammalian oncogene vs. viral) and promoter class (e.g., TATA/Inr, non-Inr), six of the eight studied promoters display TSS bubbles that are remarkably similar in length (∼10 bp) and lifetime (5–10 ps), according to the simulations. As noted, those are ‘classical’ promoters, in the sense that they represent examples of the familiar TATA box and Inr sequence elements. Among those, it might be speculated that the constitutively expressed collagen and keratocan promoters, which exhibit strong and well pronounced bubbles at the TSS, may require less assistance with DNA unwinding during transcription initiation than the less transcriptionally active, inducible gene ABF-1 [23]. PU.1 gene is another tightly regulated gene, but the experimental evidence suggests that this gene is constitutively active and is down-regulated post-transcriptionally [24],[25].

Interestingly, it has been proposed that most housekeeping genes have CpG island promoters that transcribe from multiple TSS (reviewed in [18]). In this study, the HSV-1 UL11 and the snRNA are more G/C-rich than the rest of the simulated promoters (75% and 69% G/C, respectively) and both contain CpG islands upstream of the TSS (not shown). Whether the observed broad dynamic activity across these promoters corresponds to a distinct mode of regulation through the presence of multiple TSS remains to be established. In addition to the eight promoters shown in Figure 1, we tested several promoters with very high G/C-content (80%–95%) in the TSS region. These promoters did not display any significant probability of opening at the start site (data not shown). The observed dynamic profiles of G/C-rich promoters may result from a bias of the PBD model against G/C-rich sequences, introduced by the sequence independence of the stacking potential (Eq. 1). Experimental evidence by us and also by others suggests that G/C tracks exhibit unusual base pair opening [26] and melting [27] behavior and we are currently modifying the stacking term [28] to incorporate such effects (Alexandrov et al., submitted). It should be emphasized that the PBD model performs well for ‘mixed’ sequences and a heterogeneous stacking term should not introduce significant changes in the majority of the shown profiles.

We believe that establishing the general mechanistic features of transcription initiation requires detailed understanding of both the sequence and the structure/dynamics of promoter DNA. PBD Langevin dynamic (LMD) simulations occupy a unique niche between fast bioinformatic methods and all atom simulation techniques. We have used PBD LMD to derive three different criteria describing the strand separation dynamics of promoter DNA sequences. The results suggest that the most stable dsDNA openings do not necessarily coincide with the most probable openings or with the highest average strand displacement, underscoring the advantages of proper molecular dynamic simulations. According to the simulations, each promoter exhibits distinct DNA dynamic characteristics, but the transcriptional start site is often distinguished by large, relatively stable openings in the double helix. Such local openings are likely to be recognized and engaged by the transcriptional machinery, and may then be amplified, stabilized, or suppressed by DNA-protein interactions as part of gene transcriptional regulation. Data from *in vitro* transcription experiments directly support the stable bubble requirement for DNA transcription by RNA polymerase in the absence of any transcription factors.
